# This Is My (Post) Truth, Tell Me Yours: Comment on "The Rise of Post-truth Populism in Pluralist Liberal Democracies: Challenges for Health Policy"

**DOI:** 10.15171/ijhpm.2017.58

**Published:** 2017-05-15

**Authors:** Martin Powell

**Affiliations:** Health Services Management Centre, School of Social Policy, University of Birmingham, Birmingham, UK.

**Keywords:** Populism, UK National Health Service (NHS), Post-Truth Politics, Health Policy

## Abstract

This is a commentary on the article ‘The rise of post-truth populism in pluralist liberal democracies: challenges for health policy.’ It critically examines two of its key concepts: populism and ‘post truth.’ This commentary argues that there are different types of populism, with unclear links to impacts, and that in some ways, ‘post-truth’ has resonances with arguments advanced in the period at the beginning of the British National Health Service (NHS). In short, ‘post-truth’ populism’ may be ‘déjà vu all over again,’ and there are multiple (post) truths: this is my (post) truth, tell me yours.

## Introduction

Ewen Speed and Russell Mannion^1^ have provided an interesting account of the challenges for health policy of the rise of post-truth populism in pluralist liberal democracies. The most interesting element in their discussion is a different ‘independent variable.’ While many commentators have attempted to speculate on the impact of Brexit on health policy,^2^ Speed and Mannion explore the impact of post-truth populism on health policy (ie, the ‘cause of the cause’ as populism ‘causes’ Brexit).

They rightly acknowledge that populism is one of the most contested concepts in the social sciences, but strangely seem to accept their other key term of ‘post truth’ as unproblematic. They provide a number of definitions of populism, but I can mischievously add one of my own. Populism seems to be a strange category of an ‘irregular noun,’ defined as ‘a large numbers of people having a different view to me,’ compared to democracy defined as ‘a large number of people agreeing with me.’ They discuss both right-wing (championing the ‘people’ against an elite accused of favouring a third group of their choice, usually based on unapologetic religious bigotry, racism and misogyny) and left-wing populism (championing the ‘people’ against an economically privileged neo-liberal business elite). It is not clear where Brexit fits into this spectrum, but as they stress xenophobia, it appears to be the former. However, the reasons for the Brexit vote are complex,^3^ and in some ways mirror the strange alliances of the 1970s (then) Common Market Referendum of left-wingers such as Tony Benn, Michael Foot, Barbara Castle (and a young Jeremy Corbyn, then a Labour Councillor in Haringey, now Labour leader) and right-wingers such as Enoch Powell opposing the Common Market, but for very different reasons. This means that it is difficult to know, apart from a vague ‘Leave’ vote, what Brexit populists want. However, it appears that they want more money to be spent on the National Health Service (NHS) rather than see it dismantled. They may also wish some form of ‘protectionism’ rather than the dangers of (further) ‘privatisation’ of the NHS as feared under free trade treaties such as ‘Transatlantic Trade and Investment Partnership’ (TTIP) and the ‘Comprehensive Economic and Trade Agreement’ (CETA) with Canada (which, confusingly, many ‘Left Remainers’ also oppose) (compare the Trans-Pacific Partnership).^4^

According to former Labour Minister of Health and 1970s ‘Remainer’ but 2016 ‘Leaver’ David Owen,^5^ ‘There are many people who are still unaware of how much in the 2012 NHS legislation system stems from the EU’ (p. 14). He argues that EU competition law applied to the NHS perhaps as early as 2006 (p. 115-116), drawing attention to Barbara Castle’s prediction during the 1975 Referendum campaign that a future UK government would stealthily preparing for an EU market in health (p. 116). He discusses concerns about the EU-US TTIP which had been negotiated in unprecedented secrecy (pp. 135-143) and warns of the ‘far-reaching implication for any NHS marketization because of the direction of travel within the EU towards trade links with the US based on an ever-greater application of pure market principles in the healthcare field’ (p. 139). Of course, the UK government may be stupid enough to include the NHS in any future TTIP Mark 2, but that is another story. Yet another story is that in the United States ‘Trump populism’ has called Obamacare ‘a complete and total disaster’ and supported ‘Our wonderful new Healthcare Bill’ of the American Health Care Act (abandoned in March 2017, but narrowly passed the House of Representatives in revised form in May 2017, and is now moving to the Senate). However, Republican opposition to Obamacare pre-dates Trump, with Republicans in Congress having voted more than 50 times to repeal or defund ‘Obamacare’ since 2010.^6^

In other words, it seems difficult to causally link different types of populism with impacts on health policy.

Speed and Mannion give the Oxford English dictionary (and 2016 international word of the year) definition of ‘post-truth’ as relating to or denoting ‘circumstances in which objective facts are less influential in shaping public opinion than appeals to emotion and personal belief.’ However, I am not convinced that there is a need for this new term. First, political debate has never been entirely composed of ‘objective facts.’ Second, it seems to suggest that there was a period of ‘truth’ which has been replaced by ‘post-truth.’ It is possible to argue that political language has always been characterised by appeals to emotion and personal belief. This can be summed up by writers often regarded as the essence of democratic socialism from the period at the beginning of the NHS. Aneurin Bevan famously stated that ‘This is my truth, tell me yours.’ Similarly, in 1946 George Orwell^7^ brilliantly explored the use of ‘political language,’ which in the language of his novel ‘1984’ is a ‘doubleplusgood’ essay. He stated that ‘In our time, political speech and writing are largely the defence of the indefensible’ (p. 153) and that ‘Political language […] is designed to make lies sound truthful and murder respectable, and to give an appearance of solidity to pure wind’ (p. 157).

The authors’ main concerns of challenges for health policy seem to concern inequalities and democracy. First, they claim that a populism built on ‘walls’ and fear of ‘the other’ (for Trump read Mexicans and Muslims, for Brexit read immigrants from Eastern Europe and Syrian refugees), discriminates against certain sub- sections of the population and exacerbates existing national (and global) health inequalities. The mechanisms relating to this are not fully clear, but one mechanism seems to relate to charging. They write that in the United Kingdom there have been calls to introduce charging mechanisms for ‘health tourists,’ with the effect that overseas patients are required to pay upfront for their care. This places a greater principle is at stake – ‘the introduction of a formal charging mechanism into a Beveridge based health system.’

However, this noble sentiment comes almost 70 years too late, attempting to shut the stable door long after the charging horse has bolted. The principle of the totally free health service was arguably lost in the Beveridge Report of 1942 which discussed the possibility of ‘hotel charges’ for patients in hospital, in charges for after-care which were discussed in the Standing Committee on the NHS, and in charges for prescriptions and dental and optical treatment introduced soon after the establishment of the NHS.^8^ It is also possible that a renewed focus on the charging agenda is more linked to austerity than populism or Brexit per se. For example, motions on this have been debated at BMA conferences a number of years before the announcement of the EU Referendum.

Moreover, these issues are not new. In 1952 Aneurin Bevan^9^ (p. 104-106) pointed to the ‘great deal of criticism, most of it ill-informed and some of it deliberately mischievous’ concerning the free treatment of foreign visitors by the NHS. He noted the problems of distinguishing visitors from British citizens which involved ‘means of identification’: ‘for if the sheep are to be separated from the goats both must be classified.’ He also calculated that treating visitors amounted to some 0.5% of the overall NHS budget. As any payment would have to be set against the costs of recovery, ‘happily, this is one of those occasions when generosity and convenience march together.’ He continued that when Britons go abroad they are incensed because they are not similarly treated, but he was ‘convinced’ that this will follow when other nations follow our example and have health services of their own.’ Sadly, Bevan’s crystal ball did not work on this prediction.

The populist term of ‘health tourism’ also hides a wide range of different issues such as recovering charges from individuals and governments for the cost of treatment of ‘visitors’ to arguing that ‘immigrants are swamping the NHS.’ According to the House of Commons Committee of Public Accounts,^10^ hospital trusts have had a statutory duty for over 30 years to recover the cost of treating overseas visitors who are not eligible for free care. However, it is clear that the NHS has been recovering much less than it should. Whether patients are supposed to pay for treatment depends on whether they are resident in the United Kingdom and on the type of treatment. Some treatments, including general practitioner (GP) appointments and accident and emergency care, are currently free to all patients and some patients, but most hospital care is chargeable. Trusts should charge visitors from outside the European Economic Area and Switzerland (EEA&S) directly, and report when they treat visitors from the EEA&S so that the United Kingdom can recoup charges from other member states. The NHS appears to be particularly poor at recovering charges from the EEA&S (16% in 2012-2013) compared to around 65% for outside the EEA&S. Put another way, the United Kingdom paid £674 million to other EEA&S member states in 2014–2015, but recovered only £50 million. In April 2015, new rules extended the charging regime, so that students and temporary migrants from outside the EEA&S now have to pay an immigration health surcharge as part of their visa application. The Committee concludes that the result of the EU referendum creates further uncertainty for the health system, highlighting that the Department could not explain what impact Brexit might have on the charging regime.

It has been estimated that on average immigrants benefit the country in economic terms as they tend to pay in through income tax and do not use many NHS services as they tend to be young. Moreover, it has been argued that ‘we would fall over’ without the help of the 90 000 staff from the EU who work in the social care system and the 58 000 who work in the NHS who do a brilliant job (eg, Secretary of State for Health, Jeremy Hunt^11^). However, yet again, we have been here before, as the discrimination against certain sub-sections of the population (and NHS Staff) is nothing new. According to Roberta Bivins,^12^ for decades, the BMA campaigned vigorously and vocally for the medical screening of migrants either before departure to the United Kingdom or on entry at British ports. She points to a 1965 cartoon for the ‘Daily Express’ of the figure of a large black man waiting in a GP’s surgery to represent the political problem of ‘immigration,’ a critical 1986 article in the ‘Daily Mail,’ portraying ‘immigrant women’ and their pregnancies as an unanticipated burden on the NHS, and an 1998 expose, ‘How the NHS Betrayed my Mum’ that railed, inter alia, against ‘an immigrant’ who, the outraged author claimed, received better NHS treatment. Ironically, ‘Commonwealth’ migrant staff were the mainstay of the NHS, providing treatment even to those who abused or sought to exclude them.

Turning to their second issue of democracy, Speed and Mannion state that it is a pressing necessity that health policies in liberal democracies continue to offer a breadth of coverage that ensures parity of access, based on the rigorous application of research evidence, underpinned by robust processes of democratic engagement. However, these are two very different concepts, which are sometimes mutually exclusive. It is ironic that one of the most popular institutions in Britain is one of the least democratic. Bevan went against Labour Party policy and the views of the ‘Socialist Medical Association’ (now Socialist Health Association) in basing the NHS on appointed boards rather than on elected local authorities. Many on the left have always pointed to the problem of a ‘democratic deficit’ in the NHS, and Bevan (pp. 114-115) admitted that ‘election is a better principle than selection,’ and that this might be possible in a reorganised local government system.^8^ Research evidence and democratic engagement sometimes clash head on. For example, both ‘choice and voice’ sometimes favour keeping services in smaller, local and well-loved hospitals while the evidence base often favours centralising services. Similarly, many people want (and sometimes pay directly) for complementary and alternative medicine (CAM) which do not pass the evidence threshold of randomised controlled trials.

In short, some of their key terms such as post-truth are far from new. There are different types of populism, with unclear links to impacts. In some ways, ‘post-truth populism’ may be ‘déjà vu all over again,’ and there are multiple (post) truths: this is my (post) truth, tell me yours^ [1] ^.

## Ethical issues

Not applicable.

## Competing interests

Author declares that he has no competing interests.

## Author’s contribution

MP is the single author of the paper.

## Endnotes


[1] ‘This is my truth, tell me yours’ (Aneurin Bevan, Welsh politician and founder of the NHS; subsequently used as title of album by Welsh band ‘Manic Street Preachers.’

